# Single-Pixel Imaging Based on Deep Learning Enhanced Singular Value Decomposition

**DOI:** 10.3390/s24102963

**Published:** 2024-05-07

**Authors:** Youquan Deng, Rongbin She, Wenquan Liu, Yuanfu Lu, Guangyuan Li

**Affiliations:** 1 CAS Key Laboratory of Human-Machine Intelligence-Synergy Systems, Shenzhen Institute of Advanced Technology, Chinese Academy of Sciences, Shenzhen 518055, China; yq.deng@siat.ac.cn (Y.D.); rb.she@siat.ac.cn (R.S.); wq.liu@siat.ac.cn (W.L.); 2 Shenzhen College of Advanced Technology, University of Chinese Academy of Sciences, Shenzhen 518055, China

**Keywords:** deep learning network, single-pixel imaging, singular value decomposition

## Abstract

We propose and demonstrate a single-pixel imaging method based on deep learning network enhanced singular value decomposition. The theoretical framework and the experimental implementation are elaborated and compared with the conventional methods based on Hadamard patterns or deep convolutional autoencoder network. Simulation and experimental results show that the proposed approach is capable of reconstructing images with better quality especially under a low sampling ratio down to 3.12%, or with fewer measurements or shorter acquisition time if the image quality is given. We further demonstrate that it has better anti-noise performance by introducing noises in the SPI systems, and we show that it has better generalizability by applying the systems to targets outside the training dataset. We expect that the developed method will find potential applications based on single-pixel imaging beyond the visible regime.

## 1. Introduction

Due to its function of adopting a single-pixel detector to collect the intensity of an object illuminated by a sequence of masked patterns, single-pixel imaging (SPI) is attractive in diverse applications such as ultrafast imaging [1,2], hyperspectral imaging [3,4], remote tracking [5,6], and three-dimensional imaging [7,8] for its low cost, high signal-to-noise ratio, and broadband operation [9,10,11]. The SPI techniques are especially important for wavelengths with expensive multi-pixel detectors, including terahertz [12,13,14,15], infrared [16,17,18,19], and X-ray [20,21,22,23].

In order to realize real-time SPI with high efficiency, many algorithms have been proposed or developed over the years. In 2008, Duarte et al. [24] proposed SPI based on a compressed sensing (CS) algorithm and reconstructed a 256×256 image using 1300 random patterns. However, under such a low sampling ratio, the reconstructed image suffers from relatively low quality. To improve the image quality while reducing the required acquisition time, several advanced methods based on Hadamard basis [25], Fourier basis [26], discrete cosine basis [27], and wavelet basis [28] have been developed, which leverage the completeness and orthogonality of basis patterns, as well as the sparse representation of natural images in the transform domain. Zhang et al. further compared, numerically and experimentally, the performances of Hadamard SPI (HSI) and Fourier SPI (FSI) [29], and demonstrated a 20,000 Hz projection rate using a DMD and captured 256 × 256 pixel dynamic scenes at a speed of 10 frames per second under the undersampling condition [30]. However, under the undersampling condition, these techniques truncate the spectrum by discarding the high-frequency parts, resulting in undesirable artifacts such as ringing in the reconstructed images [31].

Recently, deep learning networks have shown great potential in recovering high-quality images for SPI [32], especially when the sample ratio is exceptionally low. For example, Lyu et al. [33] developed a deep learning-based approach for ghost imaging (GI) and achieved significantly improved performance compared with the conventional CS method when the sampling ratio $S_{R}$ is down to 5%. He et al. [34] modified the convolutional neural network (CNN) so as to adapt it to GI, and achieved faster and more accurate image reconstruction than conventional GI when $S_{R}=5\%$. Higham et al. [35] proposed SPI based on a deep convolutional autoencoder network (DCAN) and recovered real-time 128 × 128 pixel video at 30 frames per second with a single-pixel camera at a sampling ratio of 2%. Rizvi et al. [31,36] developed a deep learning framework to enhance the imaging quality of four-step FSI and a fast image reconstruction framework based on DCAN for three-step FSI. They demonstrated that the proposed deep learning-based FSI outperforms the conventional FSI in terms of image quality, especially when the sampling ratio is down to 5–8%. Huang et al. [37] developed AuSamNet to optimize a sampling mask and reconstruct high-quality natural color images when the sampling ratio is as low as 7.5%. Zhu et al. [14] demonstrated an efficient terahertz SPI system incorporating deep learning networks and reduced the number of Hadamard patterns to 10% of the pixels while maintaining high image quality with acceptable signal-to-noise ratio (SNR). Stantchev et al. [38] demonstrated rapid terahertz SPI with a spatial light modulator and a convolutional neural network, and reduced $S_{R}$ to 10% while maintaining the image quality. Jiang et al. [39] proposed a novel SPI scheme for high-speed moving targets combined with a deep learning network and obtained reasonable reconstructions with a low sampling ratio of only 6%. Yao et al. [40] proposed a single-pixel classification method with deep learning for fast-moving objects and obtained feature information for classification with $S_{R}=3.8\%$. However, these data-driven networks suffer from problems such as generalizability and interpretability, which may prohibit their practical applications [41,42,43].

In order to address these problems, Wang et al. [44] proposed a physics-informed deep learning (PIDL) network and obtained better performance than other researchers using SPI algorithms at a low sampling ratio of 6.25%. We [15] demonstrated high-efficiency terahertz SPI system based on a PIDL network and reconstructed high-quality terahertz images with a significantly reduced number of measurements, corresponding to an ultra-low sampling ratio down to 1.56%, which is more efficient than the conventional HSI and FSI. However, these PIDL networks rely on GI or differential GI (DGI). It has been shown that the singular value decomposition (SVD) method can effectively enhance the fidelity and improve the imaging efficiency and quality of GI or DGI [45,46,47]. Quite recently, Cheng et al. [48] further combined the SVD-compressed GI with deep unfolding and numerically investigated its anti-noise performance at low sampling rates. However, in reports [45,46,48], the SVD algorithm was restricted to construct the encoding patterns, and the deep learning network in [48] was purely driven by data and thus also suffered from the above-mentioned problems such as generalizability and interoperability.

In this work, we propose a novel approach to realize efficient SPI based on deep learning-enhanced SVD (DLSVD). The theoretical framework and the experimental implementation are introduced, and the differences compared with conventional approaches are highlighted. We both numerically and experimentally demonstrate that the developed DLSVD outperforms the HSI, DCAN, and PIDL approaches in terms of the reconstructed image quality or the acquisition time. Remarkably, under the ultra-low sampling ratio down to 3.25%, the DLSVD can reconstruct images with improved quality over the HSI and the DCAN. The anti-noise performance and the generalization to objects of types outside the training dataset are also studied.

## 2. Theoretical Framework

Before we elaborate the DLSVD to be developed in this work, let us retrospect the classical HSI and DCAN schemes, which are adopted as references. For the HSI, one adopts Hadamard patterns to modulate the spatial intensity of optical beam illuminating the object, collects the transmitted signals with a single-pixel detector, and then reconstructs the image using the inverse Hadamard transform [29]. The Hadamard spectra comprise a series of Hadamard basis patterns in the spatial domain, $P_{H}^{u_{0},v_{0}}$, which correspond to different Hadamard coefficients and which can be generated by

(1) $$P_{H}^{u_{0},v_{0}}\left(x,y\right)=\frac{1}{2}\left[1+H^{-1}\left\{\delta (u-u_{0},v-v_{0})\right\}\right].$$

Here, $H^{-1}\left\{\cdot \right\}$ is the inverse Hadamard transform, δ(u,v) is the delta function, and $\left(u_{0},v_{0}\right)$ and (u,v) are Hadamard frequency points. For each measurement, the dot product of the object and the Hadamard basis pattern projected on it is recorded by a single-pixel detector

(2) $$C_{H}\left(u_{0},v_{0}\right)=\sum_{x=0}^{N-1}\sum_{y=0}^{N-1}P_{H}^{u_{0},v_{0}}\left(x,y\right)O\left(x,y\right),$$

where $C_{H}\left(u_{0},v_{0}\right)$ is the coefficient of the Hadamard frequency point $\left(u_{0},v_{0}\right)$, O(x,y) is the object of N×N pixels, and (x,y) represents the coordinate of the spatial domain.

By projecting a series of Hadamard basis patterns (of number *M*) onto the object, the cumulative signal can be expressed as [29]

(3) $$D_{M\times 1}=P_{M\times N^{2}}O_{N^{2}\times 1}\;.$$

The sampling ratio is defined as $S_{R}\equiv M/N^{2}$. For the full sampling, M=N×N. We can use the reshape function to transform the one-dimensional vector *D* to the two-dimensional frequency coefficient $C_{H}\left(u,v\right)$. The object’s image can be reconstructed by performing the inverse Hadamard transform $R=H^{-1}\left\{C_{H}\left(u,v\right)\right\}$.

**Figure 1:**
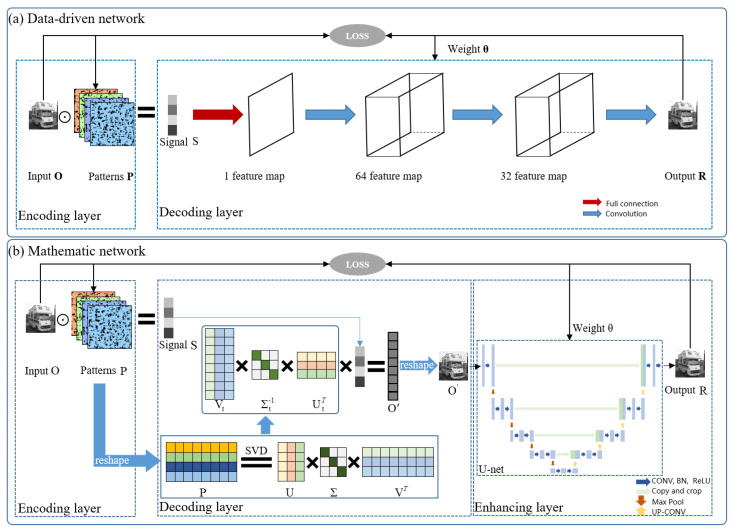
(**a**) Schematic of the DCAN adapted from [35], which includes the encoding and decoding layers. (**b**) Diagram of the developed DLSVD framework, which consists of the encoding, decoding, and enhancing layers.

The DCAN approach, as illustrated in Figure 1a, adopts autoencoders (multilayer neural networks) to encode the object, and fully connected and convolutional layers to decode objects [35]. The encoding layer simulates the signal acquisition process of SPI. When encoding object *O* with patterns *P* of number *M*, signal *S* is also collected by a single-pixel detector. The encoding process with the *i*-th pattern can be represented as

(4) $${\left(P_{i}\right)}_{N\times N}⊙O_{N\times N}=S_{i},$$

where ⊙ denotes that the two matrices are bitwise multiplied by the elements and then added together. For *M* measurements, the encoding process can be represented by the matrix as

(5) PO=S,

where *P* is a matrix of $M\times N^{2}$, of which each row corresponds to a pattern of N×N, *O* is a matrix of $N^{2}\times 1$, and *S* is a matrix of M×1.

The patterns *P* and the decoding network parameters θ are initialized randomly and then can be optimized using stochastic gradient descent to minimize the standard cost function that measures the Euclidean distance between the predicted and the desired outputs,

(6) $$\left\{P,\theta \right\}=\min_{P\in P,\theta \in \Theta ,}{∥R_{k}-O_{k}∥}^{2}.$$

The DLSVD approach developed in this work is illustrated in Figure 1b. Similar to the PIDL, the proposed method also consists of an encoding layer, a decoding layer, and an enhancing layer. The encoding process can also be expressed as Equation (5), which is the same as that of the DCAN. Distinct from the literature on SPI involving the SVD, where the SVD method was used to construct the encoding patterns [45,46,48], here, the SVD algorithm is used for decoding instead. This is also distinct from the PIDL, of which the decoding makes use of the conventional DGI algorithm.

For full sampling with $M=N^{2}$, the matrix *P* is a square matrix and can have an inverse, thus the target *O* can be reconstructed directly via

(7) $$O=P^{-1}S.$$

For undersampling condition with $M<N^{2}$, however, matrix *P* is not a square matrix but a singular matrix. For such a singular matrix, one can perform SVD to calculate its pseudo-inverse,

(8) $$P=U\Sigma V^{T}.$$

Here, *U* is an M×M orthogonal matrix whose column vectors are the eigenvectors of $PP^{T}$, i.e., $UU^{T}=I$. $V^{T}$ is an orthogonal matrix of $N^{2}\times N^{2}$ whose column vectors are the eigenvectors of $P^{T}P$, i.e., $VV^{T}=I$. Σ is a diagonal matrix of $M\times N^{2}$ with elements on the diagonal being the singular values in descending order. In order to calculate the pseudo-inverse, we further truncate the singular values to the first *K* ones, where K≤M, and then obtain

(9) $$O^{\prime }=V_{t}\Sigma_{t}^{-1}U_{t}^{T}S,$$

where $U_{t}$ is a M×K matrix with orthogonal column vectors, $\Sigma_{t}$ is a K×K diagonal matrix, and $V_{t}^{T}$ is a K×L matrix with orthogonal row vectors. $O^{\prime }$ is further reshaped into the size of the input image, N×N.

We emphasize that for the developed DLSVD approach, the decoding layer adopts the SVD method, which is a noniterative and purely mathematical algorithm, to reconstruct the image. Therefore, compared with the DCAN, which adopts data-driven convolutional neural networks for image reconstruction, the developed DLSVD approach does not suffer from the interpretability problem, as demonstrated both numerically and experimentally in this work.

In order to reduce the noises in $O^{\prime }$, we add an enhancing layer composed of a convolutional neural network. Note that there is no restriction on the choice of neural network structure, although better results may be obtained by appropriately adapting the network structure. For instance, we utilize a U-net-like architecture, comprising five down-sampling layers and five up-sampling layers, designed to handle images of varying sizes [49,50]. The reconstructed image *R* after the enhancing layer can be then expressed as

(10) $$R=U_{\theta }\left(O^{\prime }\right),$$

where θ represents the set of parameters used in the U-net.

Similar to the DCAN, the encoding patterns *P* and the parameters of the U-net enhancing layer, θ, should also be trained starting with random initialization and optimized with Equation (6). Note that for the DCAN, θ are the parameters of the decoding layer, whereas for the developed DLSVD, θ are the parameters of the enhancing layer.

## 3. Simulation Results

**Figure 2:**
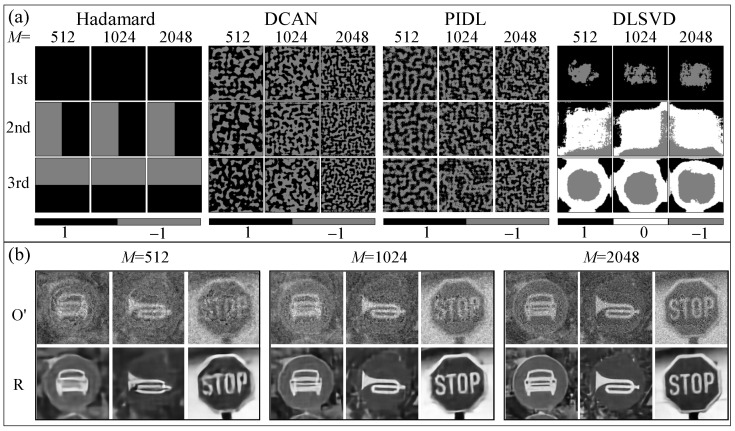
(**a**) First three typical encoding patterns for the four SPI methods with different measurements of M=512, 1024, and 2048. (**b**) Reconstructed images by the developed DLSVD with M=512, 1024, and 2048.

We first perform simulations to compare the performance of the developed DLSVD, the conventional HSI and the DCAN schemes, and the PIDL recently developed by Wang et al. [44]. Figure 2a shows the first three typical encoding patterns *P* among the *M* ones that are used in these SPI methods. For the HSI method, *M* measurements involve the first *M* encoding patterns within the well-established and well-ordered pattern pool, corresponding to the Hadamard spectra in the spatial domain [12]. In other words, for M=2048, the first 1024 patterns are exactly the same as those used for M=1024, and the first 512 patterns are the same as those used for M=512.

For the DCAN, the PIDL, and the DLSVD, however, the encoding patterns are first initialized randomly and then optimized through training, which should be performed independently for a specific measurement *M*. As a result, the encoding patterns of these approaches are different for different measurement times. We randomly select 45,000 images from the freely available ImageNet database [51] as the training set to optimize the encoding patterns and the parameters θ used in the DCAN, the PIDL and the DLSVD, and another disjoint 5000 images as the test set to test the trained network. All images are first uniformly resized to a resolution of 128×128 pixels and converted into gray-scale images, serving as the input for the networks. The learning rate is set to be 0.0002, and the number of epochs and the batch size are configured to 140 and 32, respectively. The training is carried out on an advanced gaming computer, boasting an Intel i9-12900k CPU (Intel, Santa Clara, CA, USA), 64 GB of RAM, and an NVIDIA RTX 3090 GPU (NVIDIA, Santa Clara, CA, USA).

Figure 2a further compares the first three typical trained encoding patterns of the DCAN, the PIDL, and the DLSVD with different measurements. We find that for the DCAN and the PIDL with different *M*, the trained encoding patterns all show random distributions of binary values of “1” and “−1”. As *M* increases, the patterns have higher resolution, whereas for the DLSVD with different *M*, the first/second/third trained pattern composed of ternary values of “1“, “0”, and “−1” shows pronounced similarities to its counterpart. This may correspond to the descending singular values, which are in the order of importance.

For different *M*, the trained encoding patterns of the DCAN, the PIDL, or the DLSVD are different since these patterns are first initialized randomly and then optimized through independently trained networks given the specific measurement number. Here, the networks for these approaches are defined and optimized following the details in the previous section. In other words, the optimized encoding patterns and the trained networks vary with *M*. This is distinct from the HSI, of which the first *M* patterns are taken from the well-defined pattern pool that is independent of *M*, as defined in Equation (1).

Figure 2b shows the reconstructed images $O^{\prime }$ from the decoding layer using the SVD method and the reconstructed images *R* after the enhancing layer using the U-net structure. It is clear that the SVD decoding method is efficacious and that noises can be significantly removed by the U-net enhancing layer. In other words, the SVD-based SPI method can be greatly enhanced by the DL network. Visualization 1 shows the evolution of the first encoding patterns and the reconstructed images $O^{\prime }$ and *R* after the decoding and enhancing layers, respectively, for M=1024.

**Figure 3:**
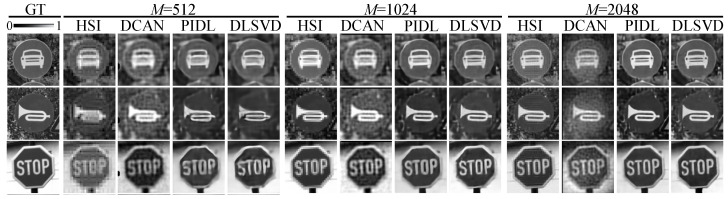
Three typical ground truth (GT) images taken from the ImageNet dataset, and reconstructed images by the four SPI methods with different measurements of M=512, 1024, and 2048.

Figure 3 shows the three typical ground truth (denoted as “GT”) images that are randomly taken from the test set within the ImageNet dataset and the reconstructed images by the HSI, the DCAN, the PIDL, and the developed DLSVD with different measurements of M=512, 1024, and 2048. Here, the traffic image subset of the ImageNet dataset is adopted for illustrating the comparison, although other target objects can also be applicable, as shown later in the experiments and on the generalizability. Simulation results show that the reconstructed images become clearer for all three methods as the number of measurements increases. For the traffic image of MotorVehicleOnly, the reconstructed images by the PIDL and DLSVD methods with M=512 clearly show the outline of the bumper and lights of the car, whereas those by the HSI and the DCAN methods require 1024 measurements to manifest the outline, and 2048 measurements to show the bumper and lights relatively clearly. Remarkably, in the reconstructed images of the PIDL and DLSVD methods with M≥1024, one can find clear details of the bumper and lights. Similarly, for the other two traffic images of HonkingNeeded and StopAndGiveWay, the PIDL and the DLSVD methods with 512 measurements can reconstruct clear outlines, whereas the reconstructed images by the HSI are muddled, and those by the DCAN suffer from relatively high background noises. The reconstructed images by the PIDL and DLSVD methods are clean and clear when the measurement number increases to 1024. In contrast, for the HSI and the DCAN, this number should be at least 2048. Given the same number of measurements, in general, the DLSVD outperforms the PIDL, followed by the HSI and the DCAN.

To quantitatively assess the quality of the reconstructed images, we adopt two metrics, PSNR and SSIM [52], which can be calculated with

(11) $$\mathrm{PSNR}=10\cdot \log_{10}\frac{255^{2}}{\mathrm{MSE}}$$

and

(12) $$\mathrm{SSIM}=\frac{2\left(u_{O}u_{R}+c_{1}\right)\left(2\sigma_{\mathrm{OR}}+c_{2}\right)}{\left(u_{O}^{2}+u_{R}^{2}+c_{1}\right)\left(\sigma_{O}^{2}+\sigma_{R}^{2}+c_{1}\right)},$$

respectively. Here, MSE is the mean square error defined as

(13) $$\mathrm{MSE}\equiv \frac{1}{N^{2}}\sum_{x=0}^{N-1}\sum_{y=0}^{N-1}{\left[O(x,y)-R(x,y)\right]}^{2},$$

where $u_{O/R}$ and $\sigma_{O/R}^{2}$ represent the average value and the variance of the input image *O* or the reconstructed image *R*, respectively, $\sigma_{OR}$ denotes the covariance of the input image *O* and the reconstructed image *R*, and $c_{1}$ and $c_{2}$ are constants used to maintain stability.

In order to compare the anti-noise performance of the four SPI methods, we further add Gaussian white noises with variance of δ to the signal. The noise level is evaluated by the signal-to-noise ratio (SNR), which can be expressed as $\mathrm{SNR}=10\cdot \log_{10}\left[\bar{{\left(S-\bar{S}\right)}^{2}}/\delta \right]$ with the $\bar{S}$ signal perturbed by the Gaussian noises. Note that Poisson noise, which is also known as photon noise or shot noise and which is prominent in low-light conditions or when using imaging sensors with low light sensitivity, is neglected in this work since it is much weaker than the Gaussian white noises.

**Figure 4:**
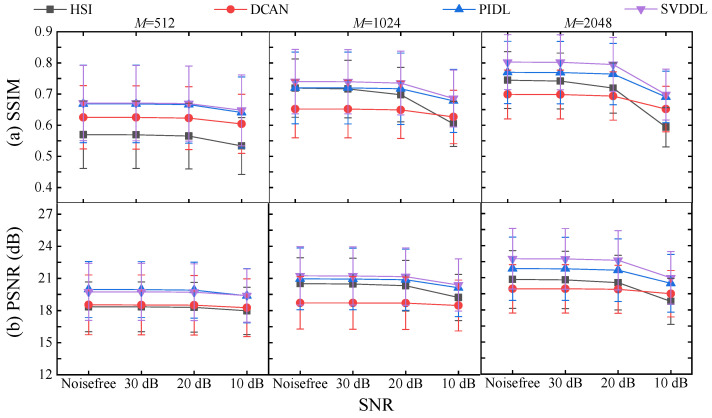
(**a**) Extracted SSIMs and (**b**) PSNRs of the reconstructed images by the four SPI methods with different numbers of measurements as functions of the ambient noise. Error bars indicate standard deviations of the test set.

Figure 4 compares the SSIM and the PSNR of the images reconstructed by the four SPI methods with different numbers of measurements as functions of the noise level. Since the calculations were performed by averaging the reconstructed images of the test set of 5000 images, the standard deviations are denoted by the error bars. Results show that for each SPI approach, both the SSIM and the PSNR increase with the number of measurements regardless of the noise level. For noise levels of SNR≤20 dB, the SSIM and the PSNR of all the four SPI methods are almost undisturbed. Given the number of measurements and under the same noise level, the DLSVD has slightly higher SSIM and PSNR than the PIDL, and larger SSIM and PSNR than the HSI and the DCAN methods. In other words, the developed DLSVD has the best anti-noise performance over the other three SPI methods.

## 4. Experimental Demonstrations

**Figure 5:**
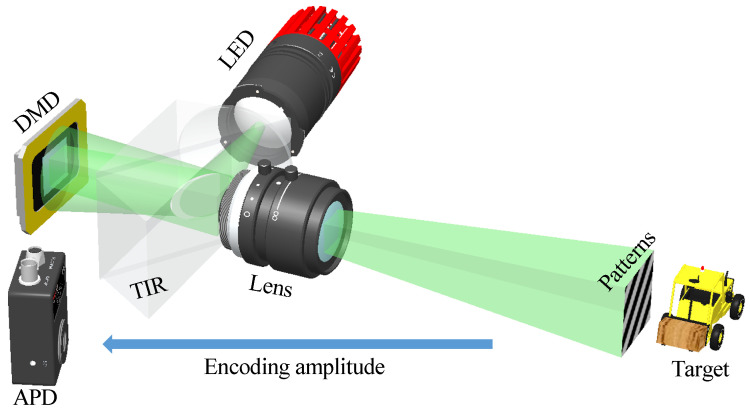
Schematic of the experimental setup.

Encouraged by the simulation results, we build an experimental setup as illustrated in Figure 5 to perform experimental validations. White light beam from a 3 W LED is directed onto a Digital Micromirror Device (DMD) using Total Internal Reflection (TIR) prisms. The patterns on the DMD at a projection rate of 100 Hz are projected onto the target through a projection lens for achieving high signal-to-noise-rate images [30]. Here, we adopt the above three printed traffic images shown in Figure 3 as the target. The reflected light from the target is captured by a single-pixel avalanche photodiode (Thorlabs APD410A2/M, Thorlabs, Newton, NJ, USA). Data are collected with the data acquisition board of National Instruments (USB6361). The DMD consists of 1024×768 micro-mirrors, each of which has a pitch size of 13.68 μm and acts as a binary reflector projecting two states: “0” and “1”, corresponding to black and white, respectively. The encoding patterns used in experiments for the four SPI methods are the same as those used in simulations, as shown in Figure 2a. In order to realize the encoding pattern element “−1” shown in Figure 2a, we employ a differential measurement method, which splits the original encoding mask with “−1” elements into two masks containing only “0” and “1” elements. Therefore, for each measurement, these masks are sampled individually, and the signals collected by the single-pixel detector are subtracted from each other for further processing.

**Figure 6:**
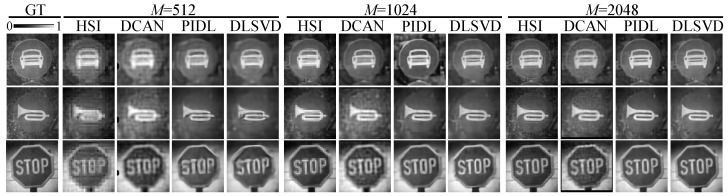
Ground truth (GT) images taken from the reconstructed ones by the HSI method with full sampling ratio ($M=N^{2}$) and experimentally reconstructed images by the four SPI methods with different measurements (M=512, 1024, 2048).

Figure 6 presents the reconstructed images by the three SPI methods with different numbers of measurements. Comparing Figure 3 and Figure 6, we find that in general, the experimental results agree well with the simulations: for all the four SPI methods, the reconstructed image quality improves as *M* increases; given the number of measurements, the developed DLSVD slightly outperforms the PIDL, which is followed by the HSI and the DCAN. Strikingly, high-quality reconstructed images can be obtained by the PIDL and the developed DLSVD even when M=512, corresponding to an ultra-low sampling ratio of $S_{R}=3.12\%$.

To quantitatively evaluate the quality of the experimentally reconstructed images, we calculate the PSNR and the SSIM by choosing the reconstructed images by the HSI at the full sampling rate of $S_{R}=100\%$, or equivalently M=128×128, as the ground truth. The results summarized in Table 1 show that the larger the number of measurements, the higher the PSNR and the SSIM all for the four SPI methods. For most cases, the proposed DLSVD method has the highest PSNR and SSIM compared with the other three SPI methods. For a few cases, the PSNR and the SSIM of the images reconstructed by the PIDL are slightly better than those recobstructed by the DLSVD.

Specifically, for M=512, the PSNR of the DLSVD is slightly larger than that of the PIDL for two images, but it is ∼1–2 dB larger than those of the HSI and the DCAN for all the three images; the SSIM of the DLSVD is slightly larger than that of the PIDL for the image of MotorVehicleOnly, but is much larger than those of the DCAN and the HSI for all the three images. As the number of measurements increases to 1024 or 2048, the differences between the PSNRs of the DLSVD and those of the other three methods further increase to ∼2–3 dB or ∼3–4 dB. On the SSIM, the DLSVD outperforms the PIDL for two images when M=1024 and for all three images when M=2048. All these quantitative metrics are consistent with the intuition from the reconstructed images by experiments (in Figure 6) as well as by simulations (in Figure 3). To offer a more comprehensive evaluation of DLSVD, we include cross-dimensional comparisons using different measurement settings. When M=1024, DLSVD achieves a mean PSNR of 24.33 dB and an SSIM of 0.7351. In contrast, when M=2048, HSI yields 22.16 dB and 0.7080, DCAN scores 22.21 dB and 0.69982, and PIDL achieves 23.56 dB and 0.7575. Notably, DLSVD outperforms HSI and DCAN, slightly surpassing PIDL in performance. In other words, we experimentally verify that, given the number of measurements, the proposed DLSVD system can reconstruct images of better quality than the HSI, the DCAN, and the PIDL methods.

The improvement in the quality of the reconstructed images is equivalent to the reduction in acquisition time, which equals the sum of the sampling time and the reconstruction time. In our experiments, all the four SPI methods share the same sampling time per pattern, which is 0.001 s. The reconstruction time is 0.02 s for the HSI, 0.004 s for the DCAN, 0.009 s for the PIDL, and 0.006 s for the DLSVD. Compared with the HSI or the DCAN, the latter of which has the shortest reconstruction time, the DLSVD can reduce the acquisition time by 50% due to half measurements required to achieve the same reconstructed image quality. Compared with the PIDL, which has comparable reconstructed image quality under the same *M*, or equivalently using the same sampling time, the DLSVD can reduce the reconstruction time by 1/3. Therefore, given the reconstructed image quality, the DLSVD is more efficient since it requires fewer measurements than the HSI and the DCAN, or has faster reconstruction time than the PIDL.

## 5. Extension to Other Objects

Although till now we have considered objects only from the ImageNet dataset, the concept and the conclusions are general, and the optimized patterns and the trained networks can be applied to other objects outside the ImageNet dataset. As an illustration, we printed four other types of objects, which are letters, cartoon patterns, and numbers. In these SPI experiments, the patterns, the networks, and the setups are the same as those used in the above experiments.

**Figure 7:**
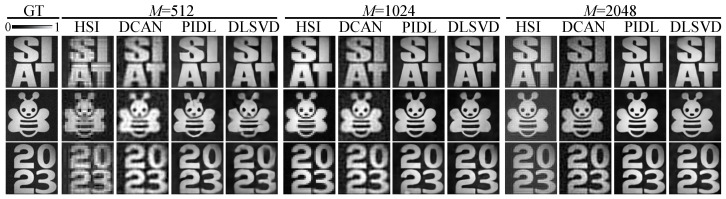
Similar to Figure 6 but with different images.

Figure 7 plots the reconstructed images by the four SPI methods. It is clear that the PIDL or the DLSVD outperforms the HSI and the DCAN methods regardless of the number of measurements. Particularly for an ultra-small number of measurements (M=512) which corresponds to an ultra-low sampling ratio of $S_{R}=3.12\%$, the images reconstructed by both the PIDL and the DLSVD have sharp edges and large SNR, whereas those by the DCAN are slightly blurred and those by the HSI suffer from relatively smaller SNR.

We further extracted the PSNRs and the SSIMs of these reconstructed images, and the results are shown in Table 2. We found that for all the four SPI systems, the quality of reconstructed images improves with *M*, that the PIDL or the DLSVD have the largest PSNRs and SSIMs compared with the HSI and the DCAN for different *M*, and that the DCAN is slightly better than the HSI. These results are consistent with those obtained with targets chosen from the ImageNet dataset (Figure 6). The exception is that the PIDL is slightly better than the DLSVD for M=512 or 1024, whereas the DLSVD outperforms the PIDL for M=2048.

Therefore, our experiment results showed that both the PIDL and the developed DLSVD SPI systems have better generalizability than the conventional HSI and DCAN systems. This is because the DL networks in both the PIDL and the DLSVD are used in the enhancing layer rather than in the decoding layer. In contrast, the DCAN takes advantage of the DL networks for decoding.

**Figure 8:**
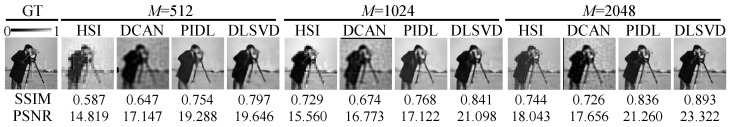
Ground truth (GT) images taken from the reconstructed ones by the HSI method with full sampling ratio ($M=N^{2}$), and numerically reconstructed images by the four SPI methods with different measurements of M=512, 1024, 2048. The values of SSIM and PSNR are extracted for each reconstructed image.

Finally, we illustrated the generalizability of the developed DLSVD with the trained patterns and networks by reconstructing complex target objects such as Cameraman. Simulation results in Figure 8 show that the developed DLSVD outperforms the other three SPI methods in terms of the image quality evaluated by the SSIM and the PNSR regardless of the number of measurements. This further validates that the developed DLSVD has the best performance in generalizability.

## 6. Conclusions

In conclusion, we proposed and demonstrated an efficient single-pixel imaging method based on DLSVD. We elaborated the theoretical framework and experimental implementation of the developed DLSVD system by comparison with the HSI, DCAN, and PIDL methods, highlighting their similarities and differences. Thanks to the encoding patterns that are optimized by the trained network, both the simulation and experimental results showed that, compared with the other three SPI methods, the developed DLSVD is more efficient in terms of the number of measurements or the acquisition time required for clear image reconstruction, and it is more robust to noises. Strikingly, even when the sampling ratio is down to 3.12%, which corresponds to 512 measurements for 128×128 pixel images, the experimentally extracted PSNRs are larger than 22 and the SSIMs are greater than 0.61 for three typical targets from the ImageNet dataset. We also demonstrated that the DLSVD outperforms the conventional HSI and DCAN systems in terms of anti-noise performance by introducing noises in the systems and of the generalizability by directly applying the SPI systems to targets outside the ImageNet dataset. Therefore, we expect this work to advance the development of SPI techniques in a variety of applications, ranging from remote sensing to microscopy beyond the visible regime.

## Figures and Tables

**Table 1:** Extracted PSNR and SSIM of the reconstructed images by the four SPI methods with different numbers of measurements. The largest values are highlighted in bold.

Measurement	Method	MotorVehicleOnly		HonkingNeeded		StopAndGiveWay	
		PSNR	SSIM	PSNR	SSIM	PSNR	SSIM
512	HSI	20.1253	0.53283	20.6722	0.57780	19.7786	0.61145
	DCAN	21.0788	0.58489	19.5797	0.64094	21.3627	0.71429
	PIDL	22.0191	0.61181	**24.2339**	**0.69649**	21.8639	**0.74317**
	DLSVD	**22.9014**	**0.61631**	24.1765	0.68647	**22.1056**	0.73374
1024	HSI	20.3322	0.59922	21.4309	0.67247	21.3050	0.70870
	DCAN	22.0833	0.60743	20.5035	0.66592	21.1787	0.72877
	PIDL	20.5111	0.64055	24.1897	0.71953	**23.9761**	**0.80488**
	DLSVD	**23.2049**	**0.67249**	**26.0226**	**0.72825**	23.7735	0.80481
2048	HSI	20.9976	0.65599	22.2591	0.71135	23.2266	0.75675
	DCAN	21.0548	0.64711	24.0583	0.68543	21.5286	0.76694
	PIDL	21.8962	0.69455	24.7208	0.74637	24.0713	0.83182
	DLSVD	**25.2250**	**0.72340**	**26.4219**	**0.76075**	**25.8388**	**0.84479**

Bold number denotes the best performance value among methods.

**Table 2:** Two indicators of experimentally reconstructed images by the four SPI methods.

Measurement	Method	Letters		Cartoon		Numbers	
		PSNR	SSIM	PSNR	SSIM	PSNR	SSIM
512	HSI	17.5826	0.56131	16.9197	0.59388	16.0013	0.47396
	DCAN	19.7788	0.64395	17.5172	0.64073	18.1696	0.63210
	PIDL	**22.8670**	**0.81414**	**21.2027**	0.73906	**22.8670**	**0.81414**
	DLSVD	21.2067	0.78155	19.8713	**0.75833**	19.5516	0.74773
1024	HSI	18.3033	0.67054	17.5807	0.62975	17.8698	0.63145
	DCAN	18.8146	0.66670	19.6356	0.68764	19.5911	0.67321
	PIDL	21.5432	**0.80972**	**21.3974**	**0.78457**	**23.8122**	**0.82897**
	DLSVD	**21.9517**	0.80432	21.0732	0.76606	23.0244	0.82127
2048	HSI	20.1011	0.71247	17.8595	0.68960	18.4002	0.69709
	DCAN	15.1268	0.65279	18.9868	0.70492	19.6354	0.70037
	PIDL	24.6053	0.83956	23.5365	0.80241	24.6324	0.83752
	DLSVD	**26.1652**	**0.84154**	**24.1226**	**0.85901**	**25.1278**	**0.86156**

Bold number denotes the best performance value among methods.

## Data Availability

Data underlying the results presented in this paper are not publicly available at this time but may be obtained from the authors upon reasonable request.
